# Radiosynoviorthesis after Surgery in the Treatment of Patients with Ankle Pigmented Villonodular Synovitis: A Case Series

**DOI:** 10.3390/jcm9020597

**Published:** 2020-02-22

**Authors:** Ioannis Iakovou, Panagiotis Symeonidis, Dimitrios Kotrotsios, Evanthia Giannoula, Christos Sachpekidis

**Affiliations:** 1 Academic Department of Nuclear Medicine, School of Medicine, University Hospital AHEPA, 54621 Thessaloniki, Greece; iiakovou@auth.gr; 2 Department of Nuclear Medicine, Aristotle University of Thessaloniki, Papageorgiou Hospital, 57006 Thessaloniki, Greece; jimkotrotsios@gmail.com (D.K.); eva_giann@hotmail.com (E.G.); 3 Department of Orthopedics, St. Luke’s Hospital, 55236 Thessaloniki, Greece; p.symeonidis@gmail.com; 4 Department of Nuclear Medicine, Inselspital, Bern University Hospital, 3010 Bern, Switzerland; 5 Clinical Cooperation Unit Nuclear Medicine, German Cancer Research Center, 69120 Heidelberg, Germany

**Keywords:** ankle pigmented villonodular synovitis (PVNS), radiosynoviorthesis (RSO), erbium-169 (^169^Er)

## Abstract

Pigmented villonodular synovitis (PVNS) of the ankle is a very rare, locally aggressive, proliferative disorder. Although surgical excision represents the standard curative treatment, the PVNS relapse rate is high. We present our study of five young athletes (range 20–36 years) with a histopathological diagnosis of PVNS of the ankle, who were treated by surgery and adjuvant radiosynoviorthesis (RSO). The operation involved either arthroscopic (four patients) or open (one patient) debridement, followed by intraarticular RSO with the radiopharmaceutical erbium-169 (^169^Er). They were evaluated with the Foot Function Index (FFI) and a visual analog scale (VAS) for pain. At a median follow up period of 47 months (range 36–54 months), all five patients reported marked pain relief with improvements in their daily activities. In particular, the median FFI decreased from 77% (range 71.0%–84.5%) pre-treatment, to 0.5% (range 0%–6%) after treatment. The median VAS score decreased from 4 (range 3–7) to 0 (range 0–1), respectively. Throughout the follow-up period, there were no major complications regarding either therapeutic intervention (arthroscopic or open debridement, RSO). Based on these results, it can be concluded that adjuvant RSO with ^169^Er following surgical excision is effective and safe in the treatment of PVNS of the ankle.

## 1. Introduction

Pigmented villonodular synovitis (PVNS) is a rare, idiopathic, locally aggressive, proliferative disorder of unknown etiology that may focally or diffusely involve the synovial tissue of the joint [1]. Most cases are monoarticular. The knee is the most commonly affected site, although the phalangeal joints, hip, ankle, and shoulder can also be involved [2]. The ankle joint is rarely affected by the disease, with an estimated incidence of 2.5% of all PVNS cases [3].

Surgical excision represents the standard curative treatment for both localized and diffuse PVNS of the foot and ankle [4,5]. However, due to the complex anatomy of the area which allows the spreading of the disease to adjacent articular spaces, the complete excision of the highly proliferative synovium is difficult to achieve, resulting in a significant risk of inadequate excision and recurrence [6,7,8]. In this context, adjuvant radiotherapy has been suggested in cases where the complete resection of the lesions cannot be accomplished. Despite the rather satisfying reported results, the documented long-term benefits of this strategy in the particular joint are still limited [6,9,10,11].

Radiosynoviorthesis (RSO) is another adjuvant therapy that has only rarely been applied in the treatment of ankle joint PVNS, with conflicting results [12,13]. However, in the published studies, the authors used the high-energy β-emitting radiopharmaceutical Yttrium-90 (^90^Y), which is characterized by a tissue penetration of 3 to 11 mm. Currently, this agent is indicated primarily for knee joints and not for joints with a narrow joint space and a thin soft tissue envelope, such as the ankle [14].

In this case series, we present our study of five young athletes suffering from PVNS of the ankle joint who were treated with synovectomy followed by adjuvant intraarticular RSO with the radiopharmaceutical erbium-169 (^169^Er).

## 2. Materials and Methods

### 2.1. Patients

Five young amateur athletes suffering from diffuse PVNS of the ankle joint underwent arthroscopic (four patients) or open (one patient) synovectomy between 01/2015 and 01/2017. There were three female and two male patients, aged from 20 to 36 years (mean age: 28.2 years). All the patients had at least one preoperative contrast-enhanced MRI. In all cases, the diagnosis was confirmed by histopathology. The present study was performed in accordance with the ethical standards laid down in the 1964 Declaration of Helsinki and all subsequent revisions. All persons gave their informed consent prior to their inclusion in the study.

### 2.2. Synovectomy

**Figure 1:**
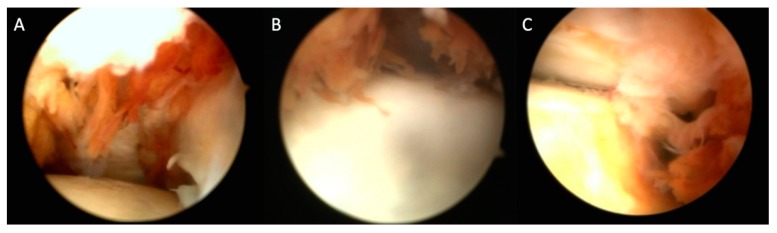
Arthroscopic image of pigmented villonodular synovitis (PVNS) of the ankle joint ((**A**,**B**) anterior arthroscopy; (**C**) posterior ankle arthroscopy) showing the villonodular tissue originating from the synovium.

An arthroscopic debridement of the synovium was performed under tourniquet with the use of an anterior or posterior ankle arthroscopy or a combination of the above (Figure 1).

In the case of a combined anterior and posterior arthroscopy, the operation was performed in two stages. In another case, the procedure was combined with a mini open approach, with an extension of the arthroscopic portal approach. Finally, in one case, the anterior tibial tendon was also involved and an anterior tendon tendoscopy was performed, followed by open debridement. The choice of the arthroscopic approach depended on the location of the affected synovium, as shown by the imaging studies. All arthroscopies were performed with a regular 3.5 mm 30° angle arthroscope and debridement was performed with a combination of 3.5 and 4.0 mm shavers. In cases where the synovium extended to the medial and lateral ankle gutters, a 2.7 or 2.9 mm shaver was also utilized. Depending on the duration of the procedure and the type of anaesthesia, the patients were treated either as a day case or with a one-night hospitalization. A regular postoperative protocol with two weeks protected weight-bearing on crutches was implemented in all cases.

### 2.3. Radiosynoviorthesis (RSO)

**Figure 2:**
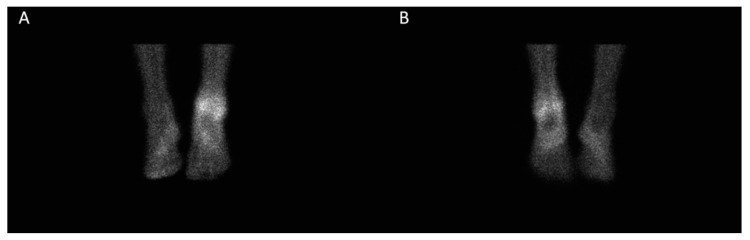
Blood pool imaging with technetium-99m-methyl diphosphonate (^99m^Tc-MDP) of the ankle joint before radiosynoviorthesis (RSO), demonstrating increased tracer uptake in the ankle joint ((**A**) anterior view; (**B**) posterior view).

All patients had a postsurgical, pre-RSO, three-phase bone scintigraphy, performed not earlier than two months after synovectomy, which confirmed an increased technetium-99m-methyl diphosphonate (^99m^Tc-MDP) uptake in the blood pool phase (Figure 2).

**Figure 3:**
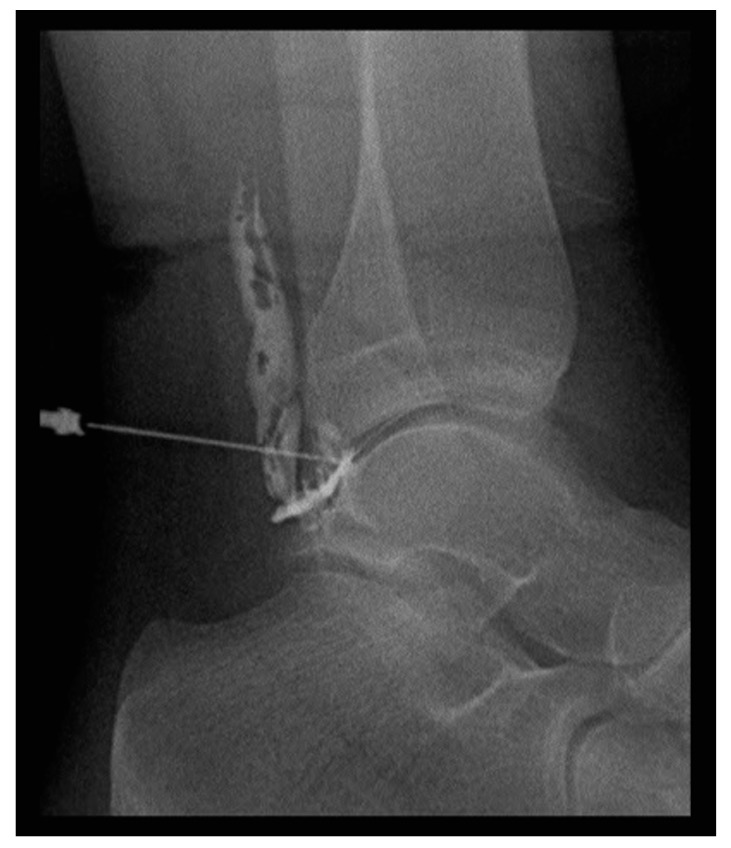
X-ray of the ankle joint confirming correct needle position before application of erbium-169 (^169^Er).

RSO was performed 3 to 5 months postoperatively. The RSO procedure was performed under strict aseptic conditions under X-ray guidance (Figure 3).

**Figure 4:**
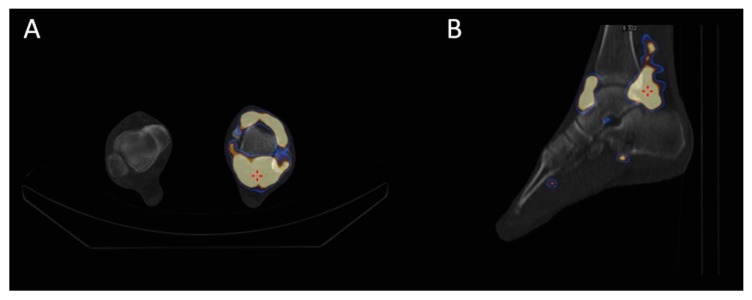
Imaging of the ankle joint performed immediately after intraarticular application of ^169^Er. Fused transaxial single-photon emission computed tomography/computed tomography (SPECT/CT) (**A**) and coronal SPECT/CT (**B**) of the ankle joint excluded potential extraarticular leakage of ^169^Er. Due to the very low intensity γ-rays of ^169^Er, imaging was performed with co-administration of 185 MBq of ^99m^Tc in order to render SPECT/CT imaging feasible.

After a local lidocaine injection for analgesia, excess joint fluid—if present—was drained, and 74 MBq of ^169^Er in a volume of less than 1 mL was injected intraarticularly into the ankle joint, followed by the administration of 20 mg of triamcinolone acetonide. Immediately after ^169^Er application, static imaging of the ankle joint with single-photon emission computed tomography/computed tomography (SPECT/CT) was performed to demonstrate the distribution of the radiopharmaceutical in the joint and exclude potential extraarticular leakage (Figure 4). The patients were instructed to avoid excessive physical activity for the following two days.

### 2.4. Follow-Up

Joint functional status and pain were assessed by the Foot Function Index (FFI) and a visual analog scale (VAS). In particular, FFI is a self-administered index consisting of 23 self-reported items divided into 3 subcategories on the basis of patient values: pain, disability, and activity limitation. The patients had to describe their foot by scoring each question on a scale from 0 (no pain or difficulty) to 10 (worst pain imaginable or so difficult it requires help). Finally, scores ranged from 0 to 100, with higher scores indicating worse pain [15,16]. FFI assessment was performed before RSO and on June 2019 at a median follow up of 47 months (range 36–54 months).

The VAS score is a unidimensional measure of pain intensity, consisting of a straight line with the endpoints defining extreme limits such as ‘no pain at all’ and ‘pain as bad as it could be’ [17]. The VAS consists of ten steps: 1—lack of any impairment to 10—total disability. Assessment was performed just before (less than twelve days) and two, six and twelve months after RSO, as well as at a median follow-up of 47 months (range 36–54 months) for all patients.

## 3. Results

The characteristics of the studied patients, as well as the data on the applied treatment, are presented in Table 1. A static scintigraphy of the ankle joint with ^99m^Tc (photopeak of 140 keV, 20% energy window) immediately after ^169^Er application confirmed the intraarticular administration of the radiopharmaceutical in the joint and excluded extraarticular leakage in all patients (Figure 4).

Successive assessment by means of FFI and VAS revealed a marked relief of the pain that limited daily activities as a percentage of the pretherapeutic joint discomfort. In particular, the FFI decreased from a median 77% (range 71.0%–84.5%) before treatment to a median of 0.5% (range 0%–6%), as assessed after communication with all patients at a median follow up of 47 months (range 36–54 months) (Table 2).

Moreover, the median pre-treatment VAS score was 4 (range 3–7) and decreased after two months to 1 (range 0–2), after six months to 1 (range 0–1), and after twelve months to 0 (range 0–1). Further, at a median of 47 months after therapy (range 36–54 months) the median VAS score was further reduced to 0 (range 0–1) (Table 3). Apart from the long duration of pain relief, all patients reported a further improvement in their daily activities.

Throughout the follow-up period, no major complications regarding either therapeutic intervention (synovectomy, RSO) were recorded. Only one patient reported pain and edema three weeks after RSO, which subsided after a week of oral non-steroid anti-inflammatory drugs (NSAIDs).

## 4. Discussion

RSO has been used for more than 60 years in the treatment of a range of refractory painful synovitis [18]. The modality is based on the local application of β-emitting isotopes with a therapeutic range of only a few millimetres in the affected joint and their subsequent phagocytosis by macrophages and other inflammatory cells in the articular cavity. The resulting local irradiation of the joint leads to necrosis of the superficial synovial layers, delaying joint destruction [19]. The therapy is minimally invasive and well-tolerated with practically no side effects, if well-performed. The main indications of RSO are rheumatoid arthritis, undifferentiated arthritis characterized by synovitis, ankylosing spondylitis, psoriatic arthritis, hemophilic arthritis, PVNS, and osteoarthritis, with synovitis as its primary manifestation [14,20].

PVNS is treated by surgical excision. However, simple resection of the lesion is associated with local recurrence rates as high as 50% [13]. RSO has been successfully applied in combination with surgical synovectomy for the treatment of PVNS mainly in the knee with the radiopharmaceutical ^90^Y [12,21,22,23]. Nevertheless, no information exists on the application of the β-emitter ^169^Er in the ankle joint. The reasons for the usage of ^169^Er lie in the post-surgical, adjuvant setting of the treatment. All patients had already undergone synovectomy, thus a large—but not whole—proportion of the hypertrophic synovium had been surgically removed. Since the main objective of adjuvant RSO was to complete the resection of the affected tissues, which were no longer markedly hyperthrophic, we chose to use a radiopharmaceutical with a maximum tissue penetration of up to 1 mm like ^169^Er and not the recommended isotope ^168^Re, which emitts β-particles that have ranges of up to 4.5 mm in tissue. Another reason for the application of ^169^Er in this patient cohort is that, during the operation (either arthroscopic or open debridement), the integrity of the joint capsule is inevitably compromised. In the postoperative period where RSO is applied, this is only partially repaired with interrupted sutures. Especially in the case of ankle arthroscopy, only the skin is repaired, with sutures leaving the joint capsule to heal by itself. This does not provide a true sealant to microleakage. In the case of posterior involvement in particular, additional facts need to be considered. Firstly, the posterior joint capsule is located deep to the retrocalcaneal fat pad, with a broad and rather loose attachment to the distal tibia. This interferes with its healing process, as the effect of tamponade from the postoperative bandages is minimized. Secondly, the Achilles tendon is unique in that it lacks a true synovial sheath. It is covered only by a thin layer of paratenon, which makes it more vulnerable to toxicity due to leakage of the RSO agent. The above reasons reinforce the choice of ^169^Er citrate, as it offers a good balance between efficacy and the minimum possible penetration.

The findings of this small case series support the use of ^169^Er RSO as an effective adjuvant to the surgical excision of PVNS in the ankle. All five patients responded to the combined treatment satisfactorily, with significant pain reduction and return to daily activities early after administration of a single dose of ^169^Er. Patient follow-up showed an essential reduction in pain intensity as early as two months after treatment and, more importantly, a lasting effect of the treatment regarding pain alleviation and ability to perform daily activities, as estimated at a median of 47 months after RSO. This result is of great importance given the young age (20–36 years) and the strenuous, everyday activity—as athletes—of the herein treated cohort.

Reports on RSO in the ankle joint with PVNS are scarce [12,13]. Shabat et al. treated three patients with diffuse PVNS of the ankle joint with debulking surgery, followed by intraarticular injection of 555–925 MBq of the high-energy β-emitter ^90^Y 6–8 weeks after the last surgery. The reported results were very satisfying regarding both safety and efficacy [12]. However, six years later, the same group reported on the treatment of seven patients with PVNS of the ankle with subtotal synovectomy followed by RSO with ^90^Y. In that paper, the authors reported extensive complications following intraarticular injection of the radiopharmaceutical, with all patients suffering from pain associated with redness and swelling of the involved ankle. More importantly, two patients developed full-thickness skin necrosis around the injection site, necessitating free muscle flap transfer, while a third patient developed a draining sinus associated with chronic severe pain [13]. These RSO-related soft tissue complications were not observed in our study. This discrepancy in toxicity of the two applied treatments can mainly be attributed to the different physical characteristics of the agents and the technique used: the β-particles emitted by ^90^Y have a longer penetration in tissue than ^169^Er, leading to the irradiation of neighboring tissues, mostly the skin, thus causing radiation-induced erythema or skin necrosis. In fact, this penetration length is the main reason that ^90^Y is nowadays indicated for large joints, like the knee, and not for medium-sized joints. Moreover, the doses applied in that study were much larger in comparison to the ones administered in our study (555 MBq vs. 74 MBq), which could also contribute to the unacceptable toxicity of the ^90^Y-RSO. Finally, following the injection of the isotope, we also administered corticosteroids into the ankle joint in order to reduce inflammation, which was not reported in the study by Bickels et al.

There are certain limitations in this study. Firstly, it is a relatively small case series. However, PVNS of the ankle is a very rare disorder, rendering the design of prospective large cohort studies extremely difficult. To our knowledge, this is the first published report of intraarticular RSO with the radiopharmaceutical ^169^Er in PVNS of the ankle following surgical synovectomy. Secondly, although all patients followed the same protocol regarding the timing and the dose of the RSO, there are certain variations in the operative management, as dictated by the different manifestations of the disease itself, leading to the use of an anterior or posterior ankle arthroscopy or a combination of the above. Nevertheless, all patients responded more than satisfyingly to treatment without variations.

## 5. Conclusions

Adjuvant intraarticular RSO with ^169^Er following arthroscopic or open excision is effective and safe in the treatment of PVNS of the ankle. Long-term follow-up is required to confirm the very encouraging results presented herein.

## Figures and Tables

**Table 1:** Characteristics of the studied patients.

Patient No	Gender	Age	Sport	Time between Synovectomy and RSO	Side-Effects	Duration of Follow-Up (Months)
1	F	20	rhythmic gymnastics	3 months	None	44
2	F	25	track and field	3 months	None	47
3	M	36	volleyball	5 months	None	54
4	F	28	track and field	4 months	None	36
5	M	32	football (soccer)	3 months	Pain, edema (3 weeks)	50

Radiosynoviorthesis (RSO).

**Table 2:** FFI before and after treatment of the studied patients.

Patient No	FFI Pre-RSO	FFI on January 2020 (Median Follow-Up: 44 Months)
1	84.5%	0.5%
2	73.0%	0.5%
3	77.0%	0%
4	71%	0.5%
5	84.5%	6%

Foot function index (FFI); Radiosynoviorthesis (RSO).

**Table 3:** VAS score before and after treatment of the studied patients.

Patient No	VAS Score Pre-RSO	VAS Score at 2 Months	VAS Score at 6 Months	VAS Score at 12 Months	VAS Score on January 2020 (Median Follow-Up: 44 Months)
1	5	1	1	0	0
2	4	3	1	1	1
3	3	1	0	0	0
4	4	1	0	1	0
5	7	6	2	1	1

Visual analogue scale (VAS); Radiosynoviorthesis (RSO).
